# The placenta and umbilical cord in prenatal care: Unseen, overlooked and misunderstood

**DOI:** 10.1111/1471-0528.17936

**Published:** 2024-08-14

**Authors:** Eric Jauniaux, Christoph Lees, Amar Bhide, Elizabeth Daly‐Jones, Deepa Srinivasan, Yinka Oyelese

**Affiliations:** ^1^ EGA Institute for Women's Health, Faculty of Population Health Sciences University College London (UCL) London UK; ^2^ Queen Charlotte's and Chelsea Hospital Imperial Healthcare NHS Trust London UK; ^3^ Fetal Medicine Unit, Department of Obstetrics and Gynecology St George's Hospital London UK; ^4^ Institute of Reproductive and Developmental Biology Imperial College London London UK; ^5^ Division of Maternal Fetal Medicine, Department of Obstetrics and Gynaecology Beth Israel Deaconess Medical School, Harvard Medical School Boston Massachusetts USA

## 1 INTRODUCTION

The prenatal diagnosis of placental anomalies was one of the first use of ultrasound imaging in obstetrics from the end of the 1960s.^1^, ^2^ Conditions such as placenta praevia and hydatidiform mole had been known for centuries to be associated with a high maternal morbidity and mortality, when undiagnosed before labour for placenta praevia or when presenting with severe anaemia and eclampsia for a hydatidiform mole. Previous attempts at imaging the placenta in utero included soft tissue radiography with radioactive isotopes injected into the maternal circulation or the amniotic cavity and pelvic angiography using radio‐opaque dyes injected into the femoral artery.

Ultrasound imaging rapidly proved more practical and safer than radiology techniques as it did not expose both mother and fetus to radiation. Rapid improvements in ultrasound resolution over the following decade made it possible to diagnose major fetal anomalies such as spina bifida,^3^ and a decade later, with the development of colour Doppler imaging, it became possible to accurately identification of small fetal vessels such as vasa praevia.^4^

Placenta praevia was originally defined using transabdominal sonography (TAS) as a placenta developing within the lower uterine segment and graded according to the relationship between the lowest placental edge and the internal cervical os.^5^ The use of high‐resolution transvaginal ultrasound (TVS) has revolutionised the diagnosis and follow‐up of placenta praevia by allowing accurate measurements of the distance between the presenting placental edge or vasa praevia and the internal os.^6^, ^7^ TVS has proven safe in patients suspected of having a placenta praevia on transabdominal ultrasound^6^ and the majority of pregnant patients in the UK who have TVS reported finding the experience acceptable.^7^

Overall, ultrasound imaging has changed the management and outcome of patients presenting with fetal congenital defects, abnormal fetal growth, multiple pregnancies and maternal obstetric disorders such as pre‐eclampsia and gestational diabetes, and has led to the development of the subspeciality in materno‐fetal medicine (MFM). Similarly, sonographers have become specialised in obstetric scanning. However, during this process, detailed ultrasound examination of the placenta and the umbilical cord has been left behind and is only superficially included in obstetric ultrasound training programmes.^8^ Furthermore, hyper‐specialisation in fetal medicine scanning has limited the exposure of both MFM and sonographer trainees to the use of TVS, which is mainly used in gynaecology and in the evaluation of patients with early pregnancy complications in specialised gynaecology clinics and early pregnancy units. In the present commentary, we address these issues and the need for the examination of the placenta and umbilical cord to be included in national training programmes on obstetric ultrasound imaging.

## 2 SCREENING AND DIAGNOSING CONGENITAL ANOMALIES OF THE PLACENTA

The incidence of placenta praevia and placenta praevia accreta has increased exponentially worldwide following a rise in the number of caesarean deliveries (CD) and in the use of artificial reproduction techniques (ART), in particular the use of in‐vitro fertilisation (IVF).^5^, ^8^ However, the UK National Screening Committee (UK NSC) has never recommended a national screening programme for placenta praevia and there is currently no systematic screening programme for placenta accreta spectrum. The NHS England fetal anomaly screening programme (FASP), last updated on the 4 of May 2023, states that the examination of placental position and amniotic fluid at the routine mid‐pregnancy (18^+0^–20^+6^ weeks of gestation) scan is not part of the NHS England FASP but is good clinical practice (https://www.gov.uk/guidance/fetal‐anomaly‐screening‐programme‐overview).

The 2021 National Institute for Health and Care Excellence (NICE) recommends offering all pregnant patients a screen for fetal anomalies and determining placental location at the routine mid‐pregnancy scan (https://www.nice.org.uk/guidance/ng201). However, it does not recommend the use of a standardised protocol for the ultrasound examination technique nor the gestational age for follow‐up examinations. A decade ago, the executive summary of a joint Eunice Kennedy Shriver National Institute of Child Health and Human Development, Society for Maternal‐Fetal Medicine, American Institute of Ultrasound in Medicine, American College of Obstetricians and Gynaecologists, American College of Radiology, Society for Paediatric Radiology and Society of Radiologists recommended that the term ‘placenta praevia’ is only used when the placenta lies directly over the internal os.^9^ For pregnancies >16 weeks of gestation, the placenta should be reported as ‘low‐lying’ when the placental edge is <20 mm from the internal os and as normal when the placental edge is 20 mm or more from the os on transabdominal or TVS. This protocol has been recommended by Royal College of Obstetricians and Gynaecologists (RCOG) Green‐top Guideline No. 27a on the diagnosis and management of placenta praevia and placenta accreta^5^ but not implemented in routine practice as many centres worldwide continue to use variable ultrasound criteria for diagnosis of placenta praevia.^8^

Placenta accreta spectrum (PAS) is a clinical diagnosis where the placenta is abnormally attached to the uterine wall at birth requiring surgical resection of the accreta area or a hysterectomy in case of extended lesions.^10^ When unsuspected at the time of delivery, attempts to manually remove accreta placental tissue can be associated with major and uncontrollable bleeding and thus ultrasound imaging plays a major role in identifying pregnant patients with a high probability of PAS at birth.^5^, ^8^ Caesarean sections increase the risk of both placenta praevia and placenta accreta in subsequent pregnancies and the risk increases with the number of previous caesarean sections.^5^, ^8^ The CD rate has increased 2–3 fold since the end of the last century in most medium and high resources countries and over 90% of PAS are now found in patients with a history of previous CD, presenting with an anterior low‐lying placenta or placenta praevia.^5^, ^8^ Patients with a placenta praevia accreta are at high risk of intra‐operative complications, in particular massive obstetric haemorrhage and should managed by an expert multidisciplinary team.^5^, ^8^

The ultrasound signs associated with PAS at birth have investigated for over three decades. A recent modified Delphi study^10^ of the ultrasound signs associated with PAS at birth has reported that consensus was reached by the expert panel that a prior history of CD, myomectomy or PAS should be the indication for detailed PAS ultrasound assessment. Targeted antenatal screening including well‐defined ultrasound signs and the precise placental position on TVS should therefore be implemented nationally for these patients so that they can be identified at the 20 weeks fetal detailed anatomy scan and referred to a specialist centre for management. The lack of formal training in ultrasound examination of patients at risk of PAS will lead to false negative cases with the corresponding higher morbidity associated with undiagnosed PAS before birth but also to false positive cases with unnecessary referral to specialist units and/or unnecessary additional surgical procedures.

## 3 SCREENING AND DIAGNOSING CONGENITAL ANOMALIES OF THE UMBILICAL CORD

A single umbilical artery (SUA) cord is of the most frequent anomalies in humans, affecting around 0.5% of pregnancies.^11^ A SUA is often found in syndromes such as aneuploidies, acardiac fetuses or sirenomelia and can explain the high perinatal morbidity and mortality of SUA when associated with major fetal organ defects. Around two‐thirds of fetuses with a SUA do not have other anatomical defects and are referred to as having an isolated SUA.^11^ A higher incidence of fetal growth restriction has been reported among fetuses with an isolated SUA and may be present without any other congenital anomalies on ultrasound examination or at birth in 10%–15% of cases.^11^ A 2‐vessel cord is included in NHS FASP handbook for the 20‐week screening scan base menu which recommends that “if this finding is seen during the scan, then locally agreed pathways should be followed” (last updated 19 February 2024). We did not identify any recommendation for the routine examination of the umbilical cord for the number of vessels at birth on the NHS England nor NICE websites but it is included in the protocol of routine medical examination of the newborn at both in NHS Wales (https://www.wisdom.nhs/anurin‐bevan‐file) and in the ultrasound examination guidelines of a few local NHS trust in England (https://www.bfwh.nhs.uk and https://www.bsuh.nhs.uk).

Abnormalities of the cord insertion have never been included in any of the obstetric ultrasound screening programmes in the UK and are only recorded at delivery in cases of stillbirth or acute intra‐partum fetal complications as part of placental histopathologic examination. A velamentous cord insertion (VCI) refers to an umbilical cord that is inserted into the membranes.^8^ VCI is found in approximately 1% of births. Around 3%–4% of patients presenting with a VCI also have a vasa praevia (VP) whereas around 2/3 of patients with a VP have a VCI.^4^, ^8^ VP has been reported to occur in around 1 in 2000 births but its prevalence is probably higher as it is often difficult to ascertain on a delivered placenta.^4^ The incidence of VCI and thus of VP is increased in multiple pregnancies and in pregnancies resulting from IVF.^4^, ^8^ There are three types of VP depending if the free vessel is connected to a VCI (type I), connected to a succenturiate or accessory lobe of the main placenta with (type II) or running in the membranes at the edge of a low‐lying placenta (type III).^4^, ^8^ When undiagnosed before delivery, VP is associated a 55% perinatal mortality and high risk of long‐term neurodevelopmental handicap in the survivors.^4^ Targeted screening of high‐risk patients (with pregnancies resulting from IVF or those presenting with a VCI or low‐lying placenta) has been shown to be efficient in reducing the mortality and morbidity of VP^12^ and general screening in recommended in the guidelines of many Western countries. In the UK, the June 2023 review by UK NSC recommends against screening for VP because it is not known how many babies are affected in the UK, how accurate the screening is, and because of the risks unnecessary early CD and false negative cases. (https://www.view‐health‐screening‐recommendations.service.gov.uk/vasa‐praevia). This recommendation is based on an external review published in 2017 by a private contractor (Costello Medical Consulting Ltd; www.costellomedical.cpm) for the NSC and does not include a discussion on targeted screening for high‐risk patients.

## 4 CONCLUSION

Anomalies of the placenta and umbilical cord can be easily screen for antenatally at the 20‐week detailed fetal ultrasound examination and their diagnosis before birth are among those most likely to prevent perinatal morbidity and mortality for both mothers and their baby. A brief web search of the many obstetric ultrasound courses and training programmes did not find on‐line or hands‐on courses that included sessions dedicated to the examination of the placenta or the umbilical cord. It also is not part of obstetric sonographer or MFM subspecialty training, apart from a cursory mention. To reduce the impact that these anomalies have on pregnancy outcomes, there is a need to integrate this topic into the MFM and obstetric sonographer curriculum and use standardised protocols to report on for these conditions, including the use of TVS.

## AUTHOR CONTRIBUTIONS

All authors contributed to the conception and writing up of this commentary.

## FUNDING INFORMATION

No funding was obtained for this study.

## CONFLICT OF INTEREST STATEMENT

The authors report no conflict of interest.

## ETHICS APPROVAL

None.

## Data Availability

Data sharing is not applicable to this article as no datasets were generated or analysed during the current study.
